# Life in a tube: morphology of the ctenostome bryozoan *Hypophorella expansa*

**DOI:** 10.1186/s40851-019-0142-2

**Published:** 2019-08-08

**Authors:** Philipp Pröts, Andreas Wanninger, Thomas Schwaha

**Affiliations:** Department of Integrative Zoology, University of Vienna, Althanstraße 14, 1090 Vienna, Austria

**Keywords:** Boring bryozoan, *Chaetopterus*, Gnawing apparatus, Space balloons, Walkerioidea

## Abstract

Bryozoa is a large phylum of colonial aquatic suspension feeders. The boring ctenostome *Hypophorella expansa* is unique and inhabits parchment-like polychaete tubes. Morphological studies date back to the nineteenth century, but distinct adaptations to this specific habitat have not been properly analysed, which prompted us to reexamine the morphology of this recently encountered species. The colony of *H. expansa* is composed of elongated stolonal kenozooids with a distal capsule-like expansion. A median transversal muscle is present in the latter, and one autozooid is laterally attached to the capsule. Unique stolonal wrinkles are embedded in the thin parts of the stolons. Single autozooids are attached in an alternating right–left succession on subsequent stolons. Polypide morphology including digestive tract, muscular system and most parts of the nervous system are similar to other ctenostomes. The most obvious apomorphic features of *Hypophorella* are space balloons and the gnawing apparatus. The former are two fronto-lateral spherical structures on autozooids, which provide space inside the tube. The latter perforates layers of the polychaete tube wall and consists of two rows of cuticular teeth that, together with the entire vestibular wall, are introvertable during the protrusion-retraction process. The apertural muscles are in association with this gnawing apparatus heavily modified and show bilateral symmetry. Adaptations to the unique lifestyle of this species are thus evident in stolonal wrinkles, autozooidal space balloons and the gnawing apparatus. The growth pattern of the colony of *H. expansa* may aid in rapid colonization of the polychaete tube layers.

**Electronic supplementary material:**

The online version of this article (10.1186/s40851-019-0142-2) contains supplementary material, which is available to authorized users.

## Introduction

Bryozoa is a phylum of aquatic colonial suspension feeders within the Lophotrochozoa, consisting of three major clades: Phylactolaemata, Gymnolaemata and Stenolaemata. The mainly marine Gymnolaemata contains two groups, the non-calcified paraphyletic ctenostomes and the monophyletic calcified cheilostomes that arose from ctenostome-like ancestors [1, 2]. Two distinct colonial growth patterns are known in ctenostomes: carnosan and stoloniferan. Carnosans show a flat, compact colony growth pattern, whereas stoloniferans possess interconnecting kenozooidal stolons that are separated via septal pore plates from attached feeding autozooids [3]. Ctenostomes show relatively little diversity with only around 350 extant species compared to over 5000 cheilostomes [2, 4]. Only a small portion of ctenostomes exhibit a boring life style [5]. This particular life style is ancient and known since the Ordovician. These are the oldest ctenostome fossils and include several now extinct genera indicating that it is a very successful strategy [5].

Among ctenostomes, there are two known strategies of a boring lifestyle: the first uses chemical substances to bore through mostly calcareous structures, such as molluscan shells, while the second uses a mechanical gnawing apparatus [5, 6]. *Hypophorella expansa* Ehlers, 1876, is a boring, walkerioidean ctenostome that has a mechanical gnawing apparatus and constitutes the only known member of the family Hypophorellidae. Its creeping stolons bear a median transverse muscle inside their distal ends, and are succeeded by one distally attached autozooid (lateral position) and two additional distal stolons (one in lateral, one in terminal position). The colony of *H. expansa* spreads in between the layers of parchment-like tubes secreted by polychaete annelids such as *Lanice conchilega* [7] and *Chaetopterus* sp. Fertilized eggs are shed into the polychaete tube lumen via a coelomopore and develop into cyphonautes larvae, which either search for a new host tube, where they settle at the innermost surface of the polychaete tube and develop further into a kenozooidal stolon [7]. The rest of the colony (autozooids and succeeding stolons) is subsequently formed via asexual budding. Once the colony is covered with a newly secreted polychaete tube layer, the gnawing apparatus of the autozooids perforate this layer towards the tube lumen, enabling the lophophores to be everted for feeding. Considering the unique habitat and lifestyle of *H. expansa*, remarkable morphological adaptations can be expected. Since the morphology of this species has not been studied since the nineteenth century [7–9], the aim of this study was to use modern morphological techniques in order to gain a better understanding of its morphology and adaptations to its unique lifestyle.

## Materials and methods

Samples of tubes of *Chaetopterus* sp. inhabited by colonies of *Hypophorella expansa* were collected by dredging at a depth of 30 m in Rovinj, Croatia. Live samples were studied on site prior to fixation. Samples were fixed in 4% paraformaldehyde in 0.1 M phosphate buffer for one hour at room temperature, rinsed 3–4 times in phosphate buffer, and stored in phosphate buffer for subsequent analysis.

**Fixed** specimens were dissected from the tubes prior to staining. The extracted parts were then transferred to a solution of 0.1 M phosphate buffer with 2% TritonX (PBT), 2% DMSO and 6% NGS for blocking and permeabilization overnight. Afterwards, samples were incubated for 24 h in a monoclonal anti-mouse acetylated α-tubulin primary antibody (dilution 1:800). The next day, the samples were rinsed thrice for 30 min with PBS and afterwards incubated for 24 h in secondary antibody goat anti-mouse Alexa Flour 568 (Molecular Probes, Eugene, OR) (dilution 1:300). Afterwards, samples were rinsed three times for around 30 min in PBS. DAPI (Invitrogen, Carlsbad, CA, USA) in a dilution of 1:120 was added to label cell nuclei; f-actin filaments were labeled with Alexa Flour 488 phalloidin (Molecular Probes, Eugene, OR) in a dilution of 1:60. Subsequently, samples were rinsed three times for 30 min and afterwards mounted on standard microscope slides with Fluoromount G (Southern Biotech, Birmingham, AL, USA). The slides were kept at 4 °C for 1–2 days prior to examination. Analysis and image acquisition was performed on a Leica SP5 II confocal laser-scanning microscope (Leica Microsystems, Wetzlar, Germany). Rendering of dataset was conducted with Amira 6.4 (Thermo Fisher Scientific, Waltham, MA, USA). Schematic drawings were done with Inkscape (inkscape.org).

## Results

### Colony structure

**Fig. 1:**
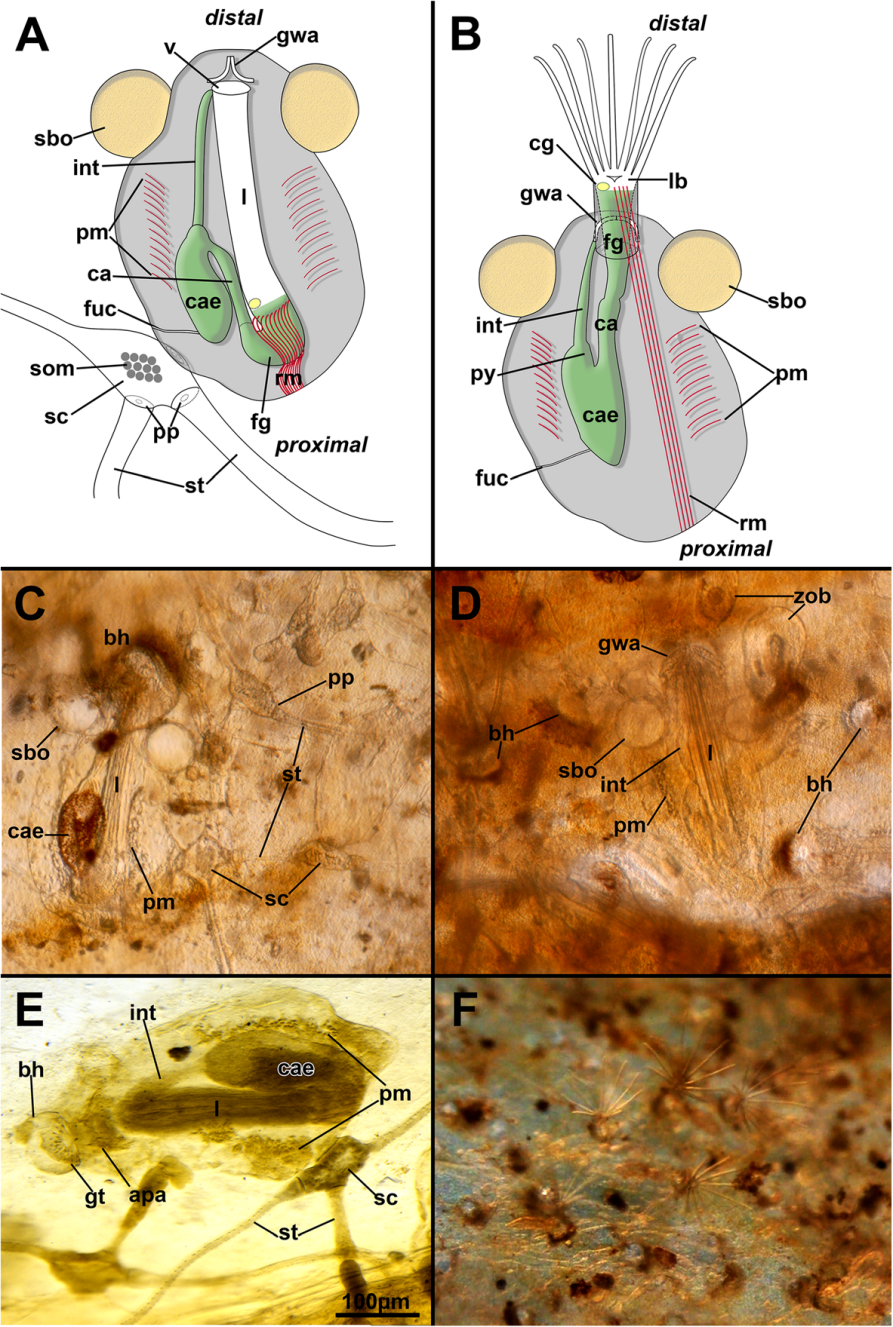
General overview of the boring ctenostome *Hypophorella expansa*. **a**, **b** Schematic drawing of autozooids attached to the kenozooidal stolons with retracted polypide (tentacles not shown) (**a**) and protruded polypide in (**b**). **c**, **d**, **f** Images of living specimens showing several morphological features when the tube layers of *Chaetopterus* are separated to thin sheets. **e** Fixed, osmified sample with the polypide stained. Abbreviations: apa – apertural area, bh – borehole, ca – cardia, cae – caecum, cg – cerebral ganglion, fg – foregut, fuc – funicular cord, gt – teeth of the gnawing apparatus, gwa – gnawing apparatus, int – intestine, l –lophophore, lb. –lophophoral base, pm – parietal muscles, pp. – pore plate, py – pylorus, rm. – retractor muscles, sbo – space balloon, sc – stolonal capsule, som – stolonal transverse muscle, st – stolon, v – vestibulum, zob – zooidal buds

**Fig. 2:**
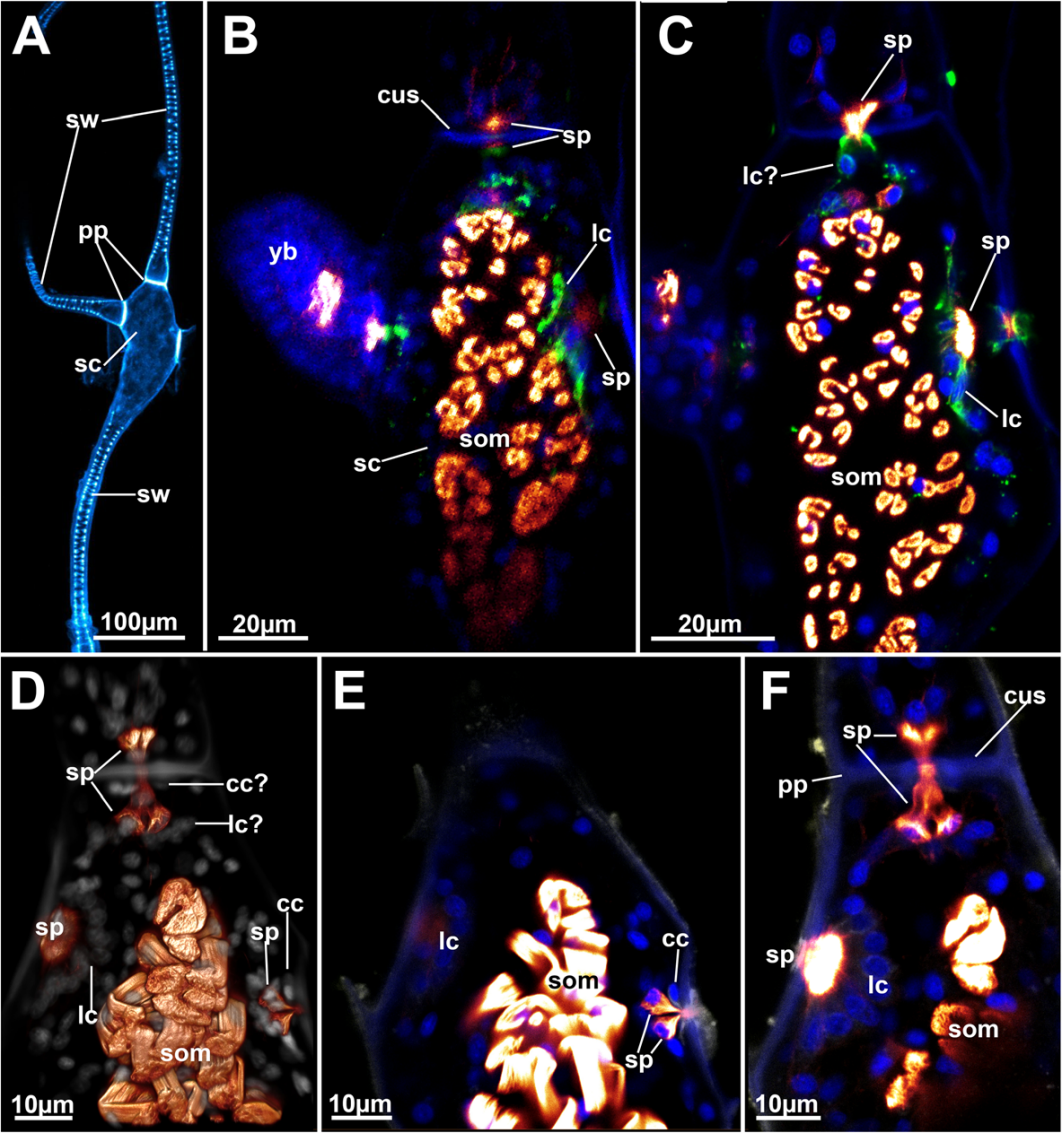
Stolonal structure of *Hypophorella expansa*. CLSM. **a** Overview using autofluorescence of a stolon. The proximal part of the stolon (lower side of the image) is thin and contains cuticular wrinkles. The distal part is widened to a capsule that also bears connections to adjacent zooids. **b** & **c** Optical sections of a stolon capsule in different planes. Staining for f-actin (glow), nuclei (blue) and acetylated alpha tubulin (green). **d** Volume rendering of a stolon capsule showing autofluorescence (grey) and f-actin (glow). **e** & **f** Optical sections of the same data set as shown in (**d**) in different section planes. Abbreviations: cc – cincture cell, cus – cuticular septum, lc – limiting cells, pp. – pore plates, sc – stolon capsule, som – stolonal transverse muscle, sp. – special cell, st – stolons, sw – stolonal wrinkles, yb – young bud

**Fig. 3:**
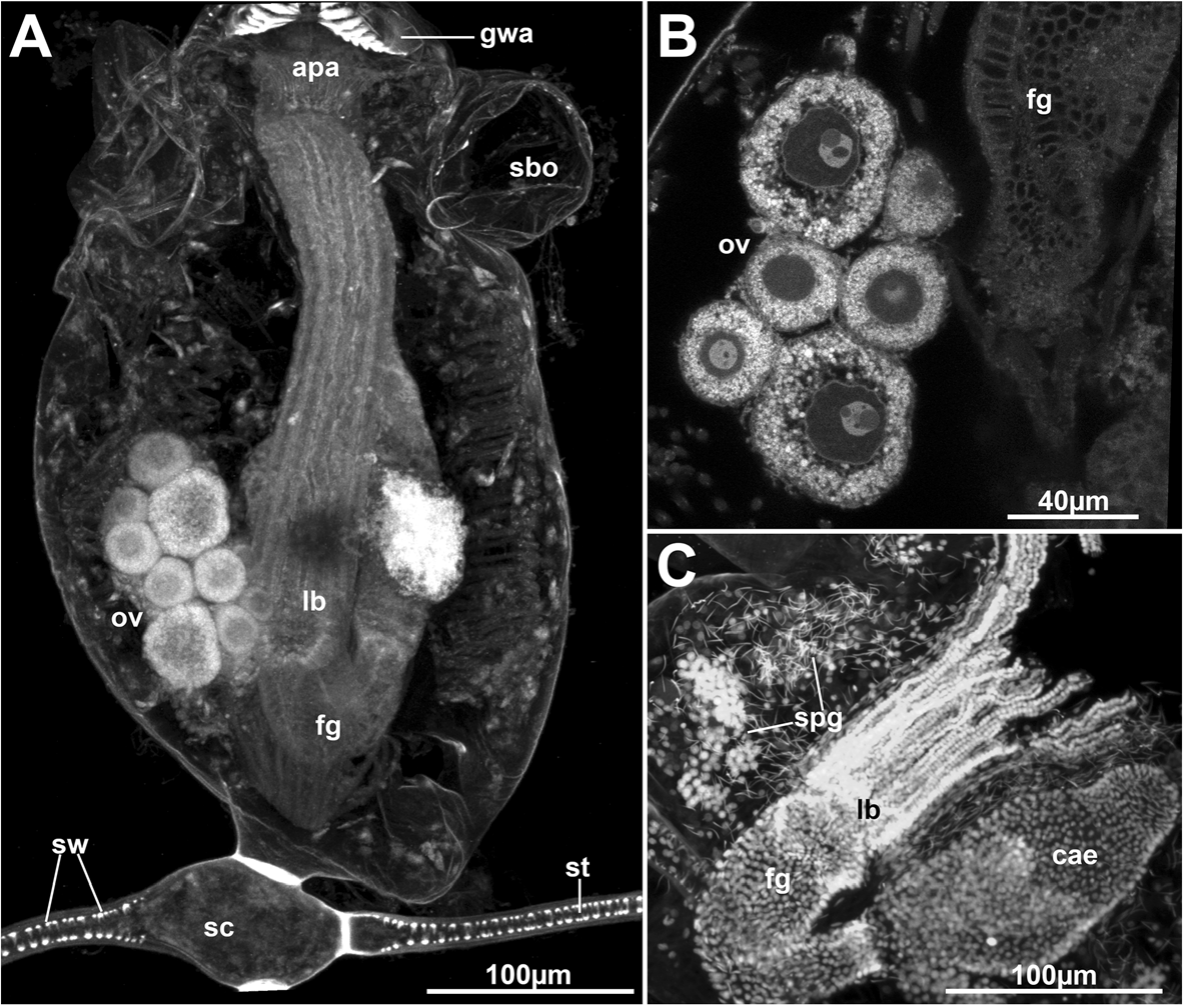
Gonads of *Hypophorella expansa*. Autofluorescence and cell nuclei staining. Confocal laser scanning microscopy (CLSM). **a** Overview of an autozooid attached to a stolon showing the laterally placed ovary with several oocytes. **b** Detail of an ovary showing nuclei with nucleoli surrounded by yolk granules in each oocyte. **c** Zooid showing laterally situated spermatogenic tissue. Abbreviations: apa – apertural area, cae – caecum, fg – foregut, gwa – gnawing apparatus, lb. – lophophoral base, ov – ovary, sbo – space balloon, sc – stolon capsule, spg – spermatogenic tissue, st – stolon, sw –stolonal wrinkles


Within the tubes of the polychaete *Chaetopterus* live colonies of *Hypophorella expansa* were found. Externally, only small pores of less than 100 μm in size are evident in the *Chaetopterus* tube walls. These boreholes of the autozooids are circular to elliptic. In live specimens, the distal margin of the boreholes was in most specimens analysed marked by a distinct brown coloration (Fig. 1c, Additional file 1: Video S1, Additional file 2: Video S2, Additional file 3: Video S3 and Additional file 4: Video S4). The bryozoan colony consists of elongated branch-like stolons and feeding autozooids attached to them (Figs. 1, 2 and 3). The body wall of both, autozooids and stolons, is composed of two basic parts: a cellular endocyst that is surrounded by a transparent, probably chitinous, ectocyst. Autozooids are oval and contain a polypide that comprises most soft tissues such as the lophophore, digestive tract and associated neuromuscular structures. They are separated from stolons via pore plates (Fig. 1). Stolons bear an autozooid at their distal lateral end, a succeeding stolon at their terminal end, and an attached lateral stolon opposite of the autozooid (Fig. 1a, c, e and 3a). The colony grows between the layers of the polychaete tube wall. A fully developed stolon possesses a slim elongated wrinkled proximal part and a distal, smooth part with a capsule-like expansion with a single attached autozooid (Figs. 1, 2a and 3a). The proximal part of a succeeding stolon directly follows the distal capsule of a preceding stolon. The lateral position of autozooids of neighboring stolons alternates from left to right in respect to the proximal-distal-axis of the stolon.

**Additional file 2: Video S2.** Overview of live autozooids of *Hypophorella expansa* showing retraction and protrusion of zooids. (MOV 191504 kb)



**Additional file 3: Video S3.** Polypide movements in an autozooid of *Hypophorella expansa*. Note also the large ovary on the right side of the zooid. (MOV 172016 kb)



**Additional file 4: Video S4.** Overview of extended lophophores of *Hyphophorella expansa*. (MP4 202235 kb)


### Stolonal structure

**Fig. 4:**
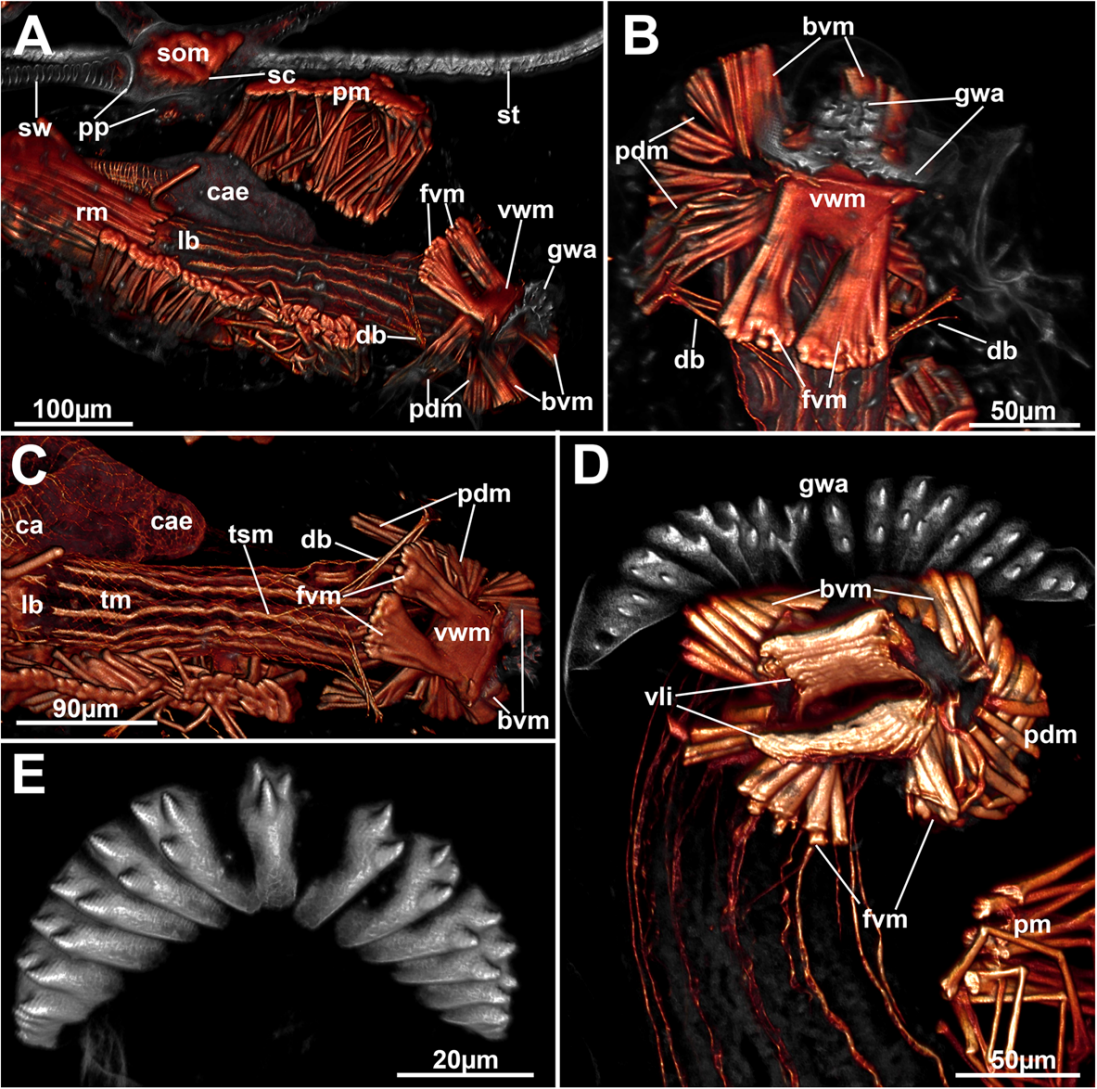
Overview of the apertural area of *Hyphophorella expansa*. Autofluorescence in gray, f-actin in glow, CLSM. **a** Oblique view of a zooid attached to the stolon capsule. **b** View from the frontal side of the aperture with unfolded vestibular wall bearing the gnawing apparatus. The rows of teeth are medially opposed. **c** Frontal overview of the muscular system of an autozooid. **d** Close-up of the apertural area with protruded gnawing apparatus. The teeth are now aligned in a single row. **e** Isolated gnawing apparatus showing autofluorscent teeth rows. Abbreviations: bvm – basal vestibular muscles, ca – cardia, cae – caecum, db – duplicature bands, fvm – frontal vestibular muscles, gwa – gnawing apparatus, lb. – lophophoral base, pdm – parieto-diaphragmatic muscles, pm – parietal muscles, pp. – pore plate, rm. – retractor muscles, sc – stolonal capsule, som – stolonal transverse muscle, st – stolon, sw – stolonal wrinkles, tm – tentacle muscles, tsm – tentacle sheath musculature, vli – vestibular lips, vwm – vestibular wall musculature

The stolons possess a cavity that, in accordance with the width of the cystid wall, widens when approaching their distal capsules (Figs. 1a, 2 and 3a). Stolon capsules possess several densely arranged muscles that run in a median position from the basal towards the frontal cystid wall: the smooth median transverse muscles (Figs. 1a, 2b-f and 4a). Their prominence and relatively dense arrangement resembles the parietal muscles of autozooids.

At the proximal part of the stolon regular wrinkles are visible at the inner side of the ectocyst (Figs. 2a, 3a and 4a). The wrinkles protrude towards the median stolon axis and show a higher density than the remaining ectocyst covering the outer side of the stolon. These wrinkles show a crescent-like shape and progress transversally from the frontal to the basal side with their endings occasionally ramifying. The wrinkling is confined to the thin areas of the stolons, and is not present at the proximal attachment or distal capsule-like site (Figs. 2a and 3a).

Each stolon is connected to both, autozooid and neighboring stolons, via a pore in a septum (Fig. 2). At the contact site of two neighboring stolons (and stolon and autozooid) the cystid walls form a transversal septum with a small central perforation, which is surrounded by a complex of three cell types that form a rosette: ‘special cells’, ‘cincture cells’, and ‘limiting cells’. These cell complexes separate body cavities of succeeding stolons and those of autozooids from stolons. Limiting cells are arranged hemispherically around the pore plates to form the outline of rosettes (Fig. 2c, d), although these cell complexes were not observed on the side of the autozooids and also appear to differ in their extent sometimes being not totally recognizable. Positive tubulin-like immunoreactivity (lir) signal has been encountered in some cases (Fig. 2c). Cincture cells seem to line the perforation of the cuticle in the rosette and turn medially where they project into the neighboring stolon or autozooid (Fig. 2d, e). Special cells plug the pore between cincture cells and limiting cells. They show f-actin and sometimes also positive tubulin-lir signal that projects through the pore between the flanking cincture cells (Fig. 2). This cell arrangement presumably serves as a communication/transport complex.

### Structure of autozooids

The autozooid is oval shaped and has a completely transparent cystid wall. It is attached proximally to the lateral side of the widened, distal stolon capsule via a small, flattened area on the basolateral side of the cystid (Fig. 1). Its polypide comprises a circular lophophore with 10–14 ciliated tentacles for food acquisition, a u-shaped gut, a nervous system with a cerebral ganglion at the anal side of the lophophoral base, and gametes in the body cavity close to the body wall (Figs. 1 and 3).

**Fig. 5:**
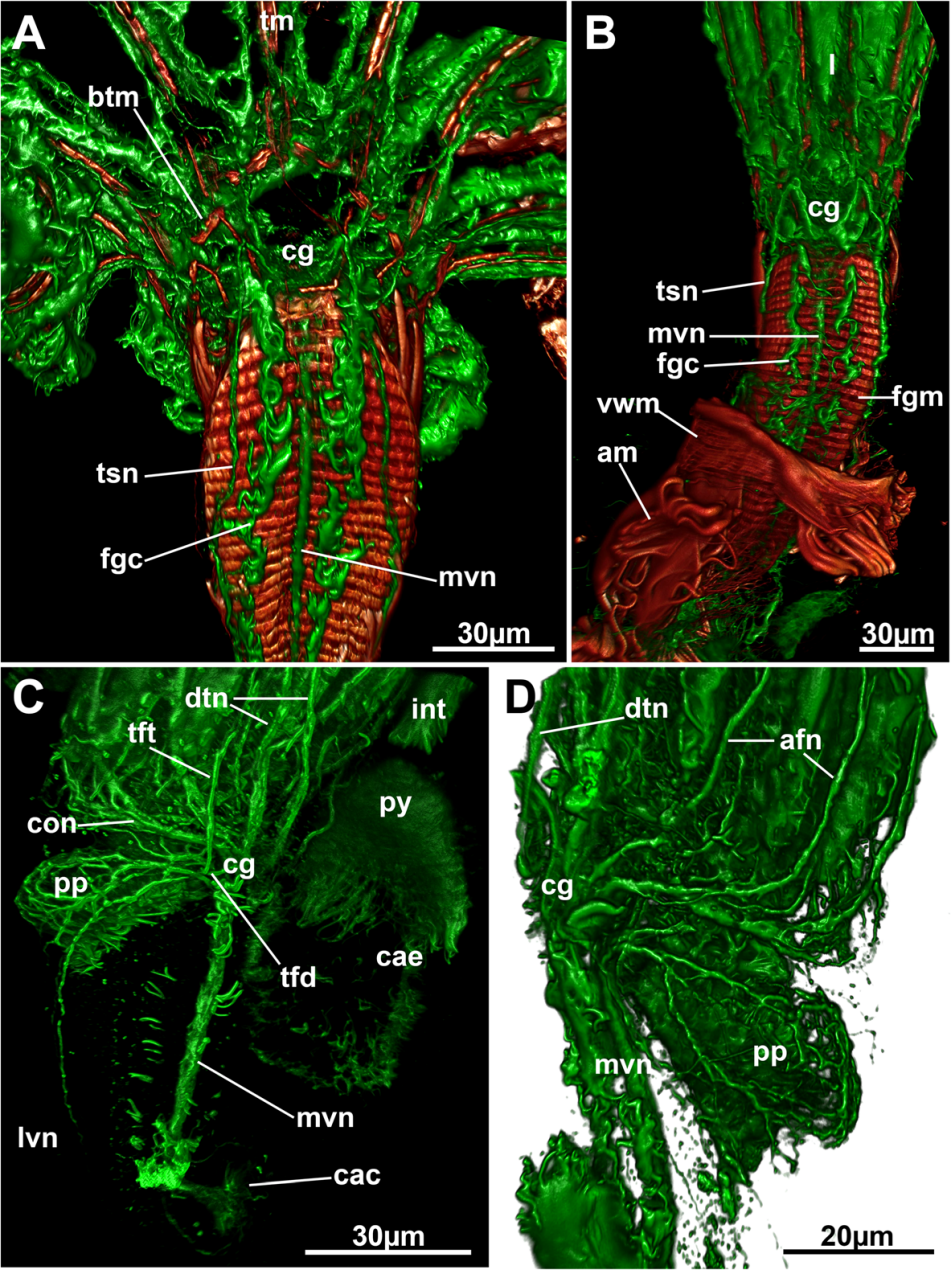
Overview of the cerebral ganglion and main neurite bundles of *Hypophorella expansa*. Acetylated alpha tubulin staining (green), f-actin (glow), CSLM, volume rendering. **a** View from the anal side of an extended polypide showing the ganglion and visceral and tentacle sheath neurite bundles. Note also the ciliary streets on each side of the medio-visceral neurite bundle. **b** Similar to A, but showing the extension of the polypide from the apertural area. **c** Oblique view of the lophophoral base showing main peripheral neurite bundles from the ganglion. **d** Oblique view of the lophophoral base showing the circum-pharyngeal plexus. Abbreviations: afn – abfrontal tentacle neurite bundle, am – apertural muscles, btm – basal transversal muscle, cae – caecum, cac – cardiac valve ciliation, cg – cerebral ganglion, con – circum-oral nerve ring, dtn – direct tentacle sheath neurite bundle, fgc – foregut ciliation, int – intestine, l – lophophore, lvn – latero-visceral neurite bundle, mvn – medio-visceral neurite bundle, pp – peripharyngeal plexus, py – pylorus, tfd – trifid nerve, tft – distal branch of trifid nerve, tm – tentacle muscles, tsn – tentacle sheath neurite bundle, vwm – vestibular wall musculature

The distolateral parts of each autozooid bear two spherical cystidial structures, the space balloons (Figs. 1 and 3a), which are located on the frontal side of autozooids and separate newly deposited tube layers of the annelid from the frontal side (the one facing the inner tube layer and the orifice of the autozooid) of the zooid. Thus, they act as wedge between the tube layers of the annelid. Both, ectocyst and space balloons, show an acellular cuticle that is lined by an epidermal layer, which is thinner inside the space balloons. Pores that connect the fluid of the space balloons with the rest of the autozooidal body are not present. The aperture or orifice is located medially between the space balloons. The distal and basal side of the vestibular wall carries a bipartite gnawing apparatus that consists of several ridges with teeth at their tips (Figs. 1a, b, 3a and 4). The protrusion-retraction process typical of all bryozoans is responsible for the movement of the gnawing apparatus (see below). When the polypide is retracted, the vestibular wall continues proximally into the tentacle sheath, which continues into the lophophoral base. At the latter, the mouth opening is situated and leads into the foregut, pharynx and esophagus. Internally, the pharynx shows distinct ciliation only at the mouth opening, which is lacking entirely in its proximal part and in the esophagus. Several zooids show two rows of cilia along the peritoneal lining on the anal side of the foregut (Fig. 5a, b). These project into the body cavity below the lophophoral base.

**Fig. 6:**
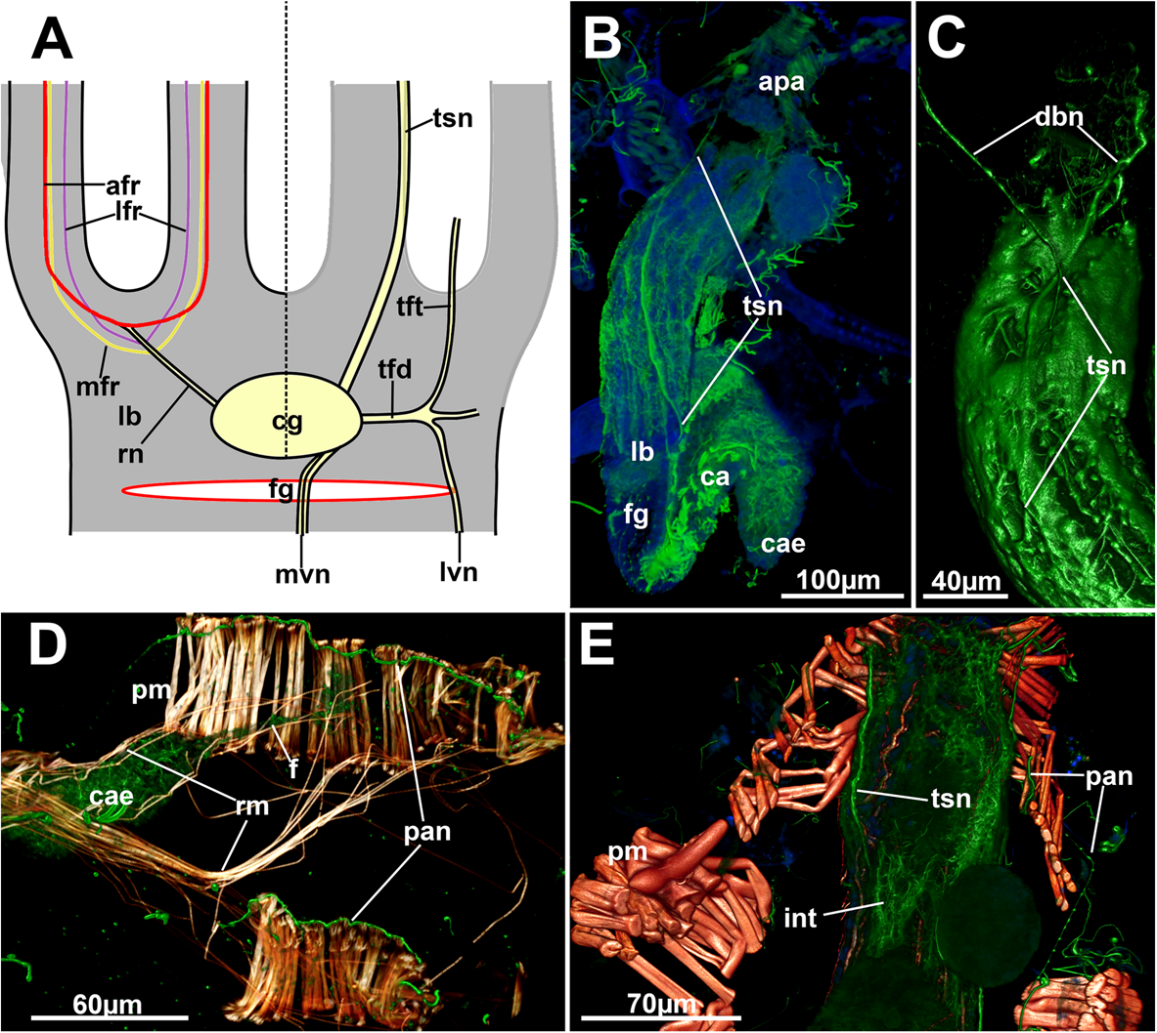
Innervation of the tentacle sheath, apertural area and body wall of *Hypophorella expansa*. Acetylated alpha tubulin staining (green), f-actin (glow), nuclei (blue), CSLM, volume rendering. **a** Schematic drawing of the lophophoral base from the anal side showing peripheral innervation on the right side, and the anal tentacle innervation above the ganglion on the left. **b** Lateral view of a retracted polypide similar as in A. **c** Detail of neurite bundles branching from the tentacle sheath neurite bundle into the duplicature bands. **d** Parietal innervation on the frontal side of the parietal musculature. **e** Close-up showing tentacle sheath neurite bundle and parietal innervation. Abbreviations: afr – abfrontal neurite bundle, apa – apertural area, ca – cardia, cae – caecum, cg – cerebral ganglion, db – duplicature band, dbn – neurite bundle along duplicature bands, f - funiculus, fg – foregut, int – intestine, l – lophophore, lb. - lophophoral base, lfr – latero-frontal neurite bundle, lvn – latero-visceral neurite bundle, mfr. – medio-frontal neurite bundle, mvn – medio-visceral neurite bunde, pan – parietal nerve, pm – parietal musculature, rm. – retractor muscle, rn – radial nerve, tfd – trifid nerve, tft – distal branch of trifid nerve, tsn – tentacle sheath neurite bundle

The esophagus terminates with a cardiac valve, which shows a distinct ring of cilia (Fig. 5c) and marks the beginning of the tubular cardia. The latter continues into a bulbous caecum (Fig. 1). At its proximal end, a very delicate funiculus extends proximally through the body cavity and inserts at the zooidal wall (Fig. 6d). The caecum shows regular short cilia and continues distally into a highly ciliated cup-like pylorus with a distally succeeding elongated intestine. The intestine shows a strong ciliation in its lumen (Fig. 5c) and terminates with an anus at the distal end of the tentacle sheath (Fig. 1).

In some of the autozooids, developing oocytes and sperm were observed. Spermatogenic tissue is situated laterally at the inner proximo-lateral side of the cystid wall (Fig. 3c). Early spermatogonia form round clusters. Free-floating spermatocytes were observed throughout the entire body cavity. The ovary is positioned laterally in the zooid, medially of the parietal musculature (Fig. 3a, b). Ovaries and testes were not observed simultaneously in the same autozooid.

### Structure and function of the gnawing apparatus

**Fig. 7:**
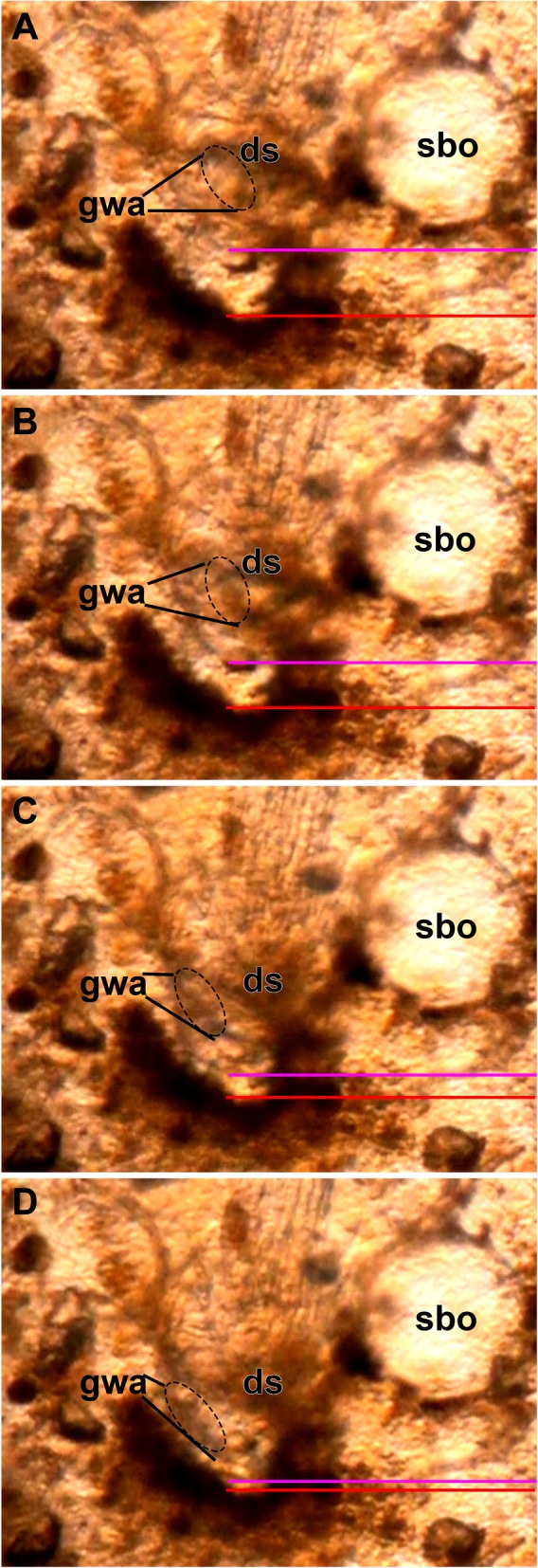
Sequence of images (from **a**–**d**) taken from Additional file 1 Video S1 showing the eversion of the vestibular wall bearing the gnawing apparatus of *Hypophorella expansa*. The red line marks the distal border of the borehole created by the bryozoan. The pink line shows the distal border of the vestibular wall as it moves closer to the border of the borehole. Note that the gnawing apparatus is discernable by its distinct brownish appearance. Abbreviations: ds – diaphragmatic sphincter, gwa – gnawing apparatus, sbo – space balloon

**Fig. 8:**
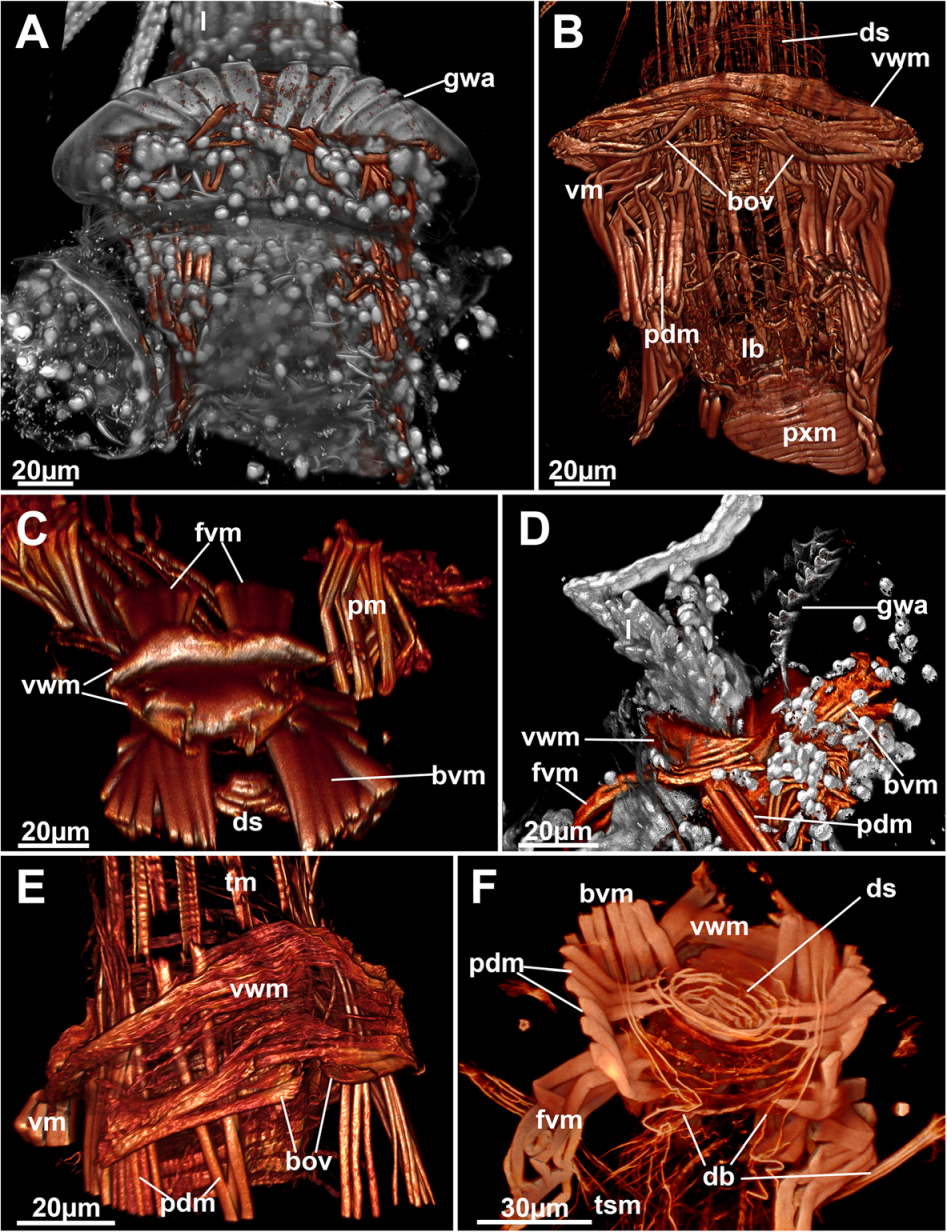
Detail of the apertural area and musculature of *Hypophorella expansa*. f-actin (glow), nuclei and autofluoescence (gray), CSLM, volume rendering. **a** Basal view of the apertural area showing a partially protruding polypide and an everted gnawing apparatus. **b** Same as in A, showing the musculature only. **c** View of the bilateral arrangement of the vestibular wall muscles in form of ‘vestibular lips’. **d** Lateral view of the partially protruded lophophore and gnawing apparatus. **e** Detail of the basal side of the vestibular wall muscles and associated apertural muscles. **f** Basal view of the apertural area showing the diaphragmatic sphincter. Abbreviations: bov – open bundles of the vestibular wall muscles, bvm – basal vestibular muscles, db – duplicature band, ds – diaphragmatic sphincter, fvm – frontal vestibular muscles, gwa – gnawing apparatus, lb. – lophophoral base, pdm – parieto-diaphragmatic muscles, pxm – pharyngeal ring muscles, tm – tentacle muscles, vm – vestibular muscles, vwm – vestibular wall muscles

The gnawing apparatus and also the space balloons are the most evident apomorphic autozooidal features associated with the boring lifestyle of *Hypophorella expansa*. Live observations of the protrusion-retraction process show that the entire apertural area unfolds distally towards the prospective, or already bored, hole in the polychaete tube wall (Fig. 7, Additional file 1: Video S1, Additional file 2: Video S2, Additional file 3: Video S3, and Additional file 4: Video S4). In retracted zooids, the gnawing apparatus consists of a series of teeth located on the disto-basal vestibular wall (Fig. 4). Both lateral rows are approximately c-shaped abutting arches with the tips of the teeth pointing inwards (Figs. 4b, d, e; 7a and 8a). The distance from the retracted zooid towards the borehole ranges around 30 μm. During the protrusion process, which is effectuated by the parietal muscles, the inverted vestibular wall unfolds fronto-distally towards the distal margin of the borehole. Simultaneously the two rows of teeth rearrange to form a semi-circle at the distal margin of the zooid (Fig. 7a–d). In protruding zooids, the teeth are pointed outwards.

### Apertural musculature

There are three different sets of muscles associated with the apertural area (Figs. 4 and 8): (1) muscles in the epithelial linings that are present as vestibular wall muscles and the diaphragmatic sphincter (Figs. 4a–d and 8b–e). The former are dense circular muscles at the border of the vestibular wall towards the diaphragm. These are laterally discontinuous and thus from a frontal and basal ‘lip’ of thick muscle bundles (Figs. 4d and 8c, e). On the basal lip the marginal bundle of these muscles is medially towards the cystid wall interrupted, too. In some cases, the median ends of these bundles are slightly bent towards the gnawing apparatus (Fig. 8a, b, e). The diaphragm with its sphincter is located basally of the vestibular wall muscles and has a series of smooth, circular fibres (Fig. 8b, c, f). (2) Eight thick muscle bundles arranged in two groups that attach to the vestibular wall or the area of the diaphragmatic sphincter. Each group consists of four bundles. The first are two prominent frontal and basal muscles that extend from the body wall to the lateral margin of the vestibular wall muscles, the parieto-vestibular muscles. The second are four bundles that originate from the basal side of the zooid and extend to the area of the diaphragmatic sphincter (parieto-diaphragmatic muscles). One pair originates more proximally, the other one more distally (Figs. 4a-d and 8). (3) Four peritoneal bands, the duplicature bands, supplied with longitudinal muscles fibres extend from the distal area of the tentacle sheath and insert at the distal body wall. Two of the bands are located frontally and insert in the area of the space balloons. The second pair is located basally and traverses below the diaphragmatic sphincter to the distal body wall (Figs. 4a–c and 8F).

Regarding the apertural muscles, the clearest distinction between a retracted and protruded condition of the polypide is the compressed and closed condition of the vestibulum and the contracted prominent vestibular muscles that also contract and fold the gnawing apparatus. In zooids with protruded polypides, the fibres of the vestibular muscles stretch and allow the dislocation of the diaphragmatic sphincter and tentacle sheath above the vestibular wall (Fig. 8b, e). The corresponding parieto-diaphragmatic muscles pass through the ring of vestibular wall muscles. The parieto-vestibular muscles are shifted laterally with the expansion of the vestibular wall itself.

### Remaining autozooidal musculature

#### Tentacle sheath muscles, parietal muscles, and retractor muscles

**Fig. 9:**
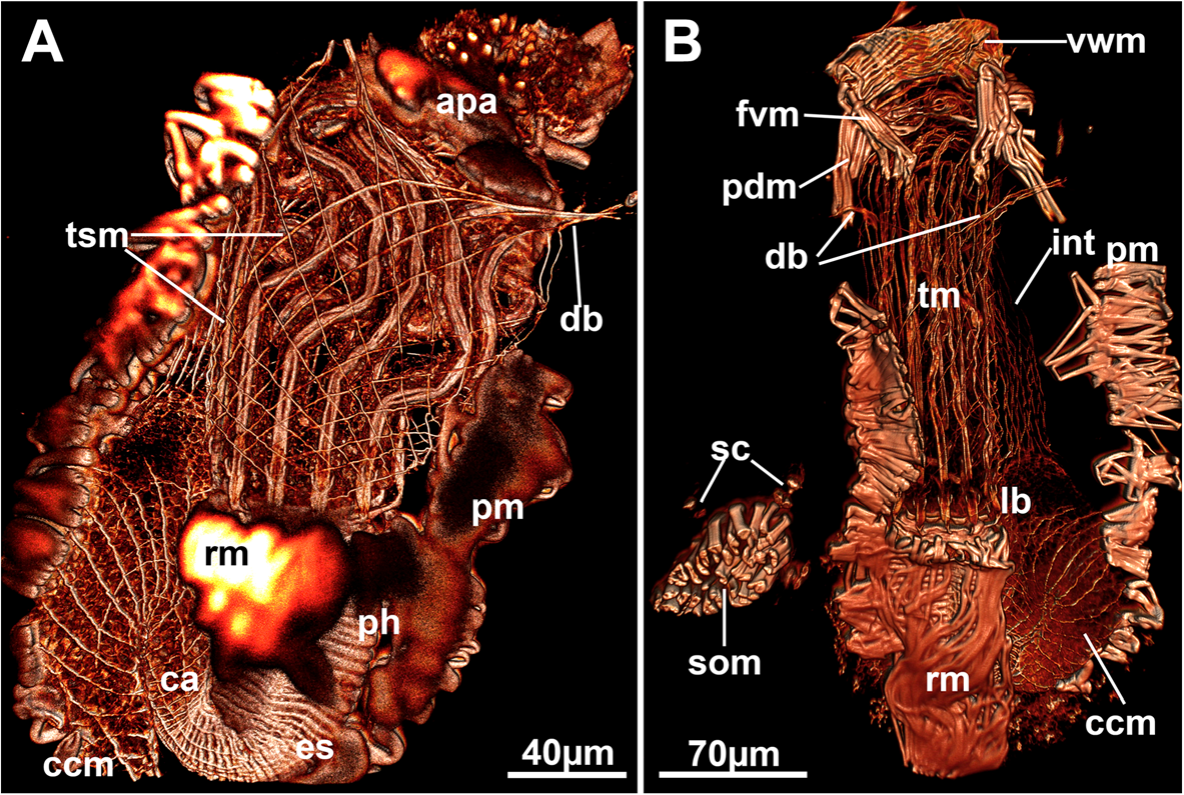
General overview of the muscular system of *Hypophorella expansa*. f-actin staining, CLSM. **a** Overview of an autozooid showing musculature of the caecum and the basket covering the tentacle sheath. **b** Autozooid showing broad parietal musculature and longitudinal muscle fibres of the intestine. Abbreviations: apa – apertual area, ca – cardia, ccm – caecal musculature, db – duplicature band, es – esophagus, fvm – frontal vestibular muscles, int – intestine, lb. – lophophoral base, pdm – parieto-diaphragmatic muscles, ph – pharynx, pm – parietal muscles, rm. – retractor muscles, sc – stolonal capsule, som – stolonal transverse muscle, tm – tentacle muscles, tsm – tentacle sheath muscles, vwm – vestibular wall musculature

The tentacle sheath possesses a net of diagonal muscles that covers the entire tentacle sheath from the lophophoral base towards its distal margin. At the latter, the diagonal fibres bundle into the muscles of the four duplicature bands (Figs. 4c and 9a).

**Fig. 10:**
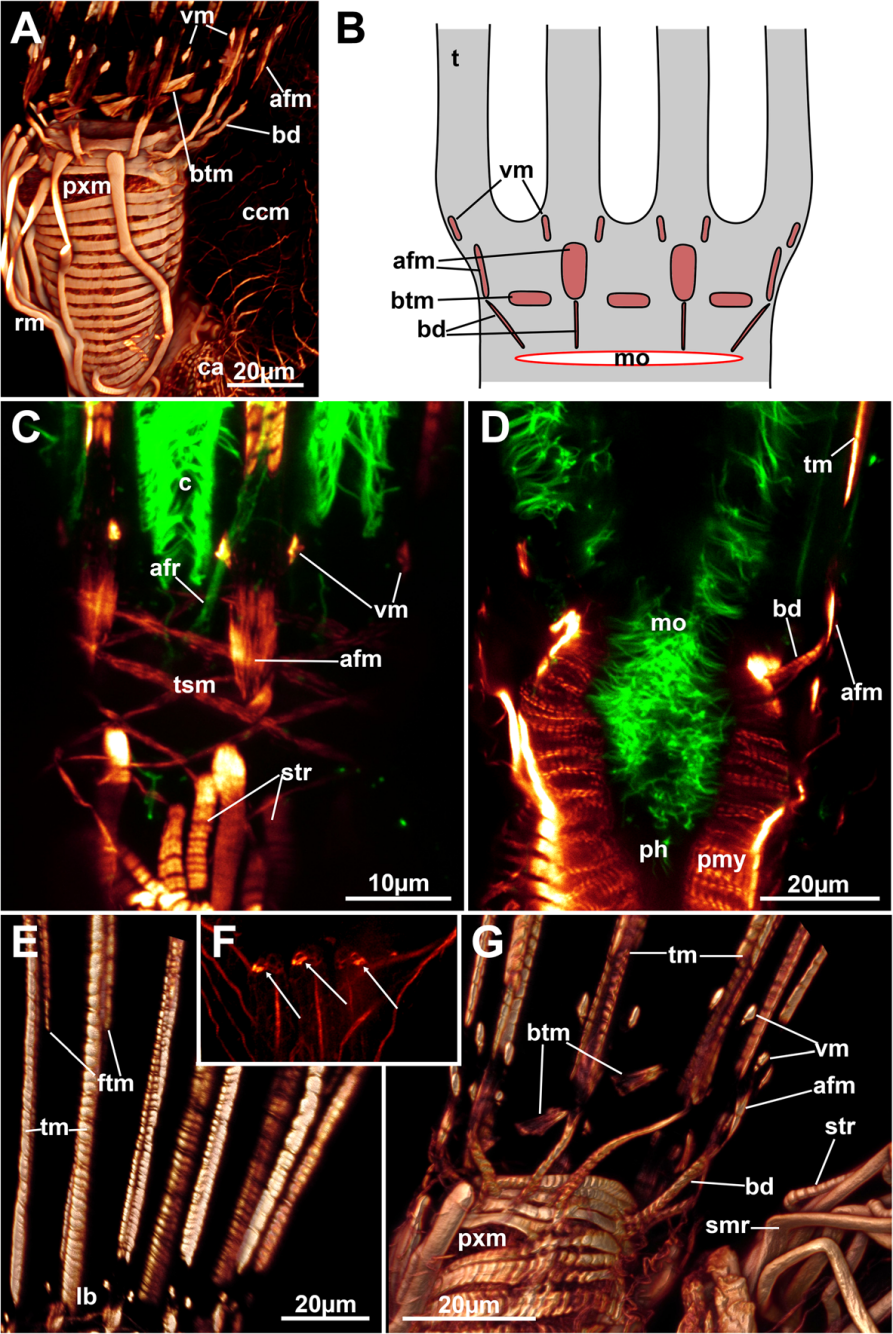
Musculature of the lophophore of *Hypophorella expansa*. F-actin staining (glow), acetylated alpha tubulin (green). CLSM. **a** Lateral view of the lophophoral base and foregut. Volume rendering. **b** Schematic drawing of the main muscular parts of the lophophoral base. **c** Optical section showing diagonal tentacle sheath muscles and abfrontal lophophoral base muscles. Note also the distinct striation of some retractor muscle fibres. **d** Optical section through the foregut showing the myoepithelium of the pharynx. **e** Lateral view of longitudinal tentacle muscle bands showing the more distally located start of the frontal muscle bands. Volume rendering. **f** Close-up of tentacle tips showing distinct circular, sphincter-like muscles (arrows). Maximum intensity projection. **g** Close-up of lophophoral base muscles. Lateral view. Volume rendering. Note the striation of buccal dilatators and the mixed, smooth and striated, forms of retractor muscle fibres. Abbreviations: afm – abfrontal lophophoral base muscles, afr – abfrontal neurite bundle, bd – buccal dilatators, btm – basal tranversal muscle, c – cilia, ccm – caecal muscles, ftm – frontal tentacle muscles, lb. – lophophoral base, mo – mouth opening, ph – pharynx, pmy – pharyngeal myoepithelium, pxm – pharyngeal ring muscles, rm. – retractor muscles, smr – smooth retractor muscle fibres, str – striated retractor muscle fibres, t – tentacle, tm – tentacle muscles (abfrontal), tsm – tentacle sheath muscles, vm ‘v’-shaped lophophoral base muscles

The parietal muscles are situated laterally on both sides in the autozooidal body cavity and consist of a continuous row of densely arranged, smooth and transverse muscle bundles that emanate from the basal to the frontal cystid wall (Figs. 1, 4a and 9). Prominent retractor muscles extend from the proximal cystid wall towards the lophophoral base. A few bundles additionally attach to the foregut. The fibres are either smooth or striated, commonly both occurring in adjacent fibres in *Hypophorella* (Fig. 10a, c, g).

#### Lophophoral base and tentacle musculature

Surrounding the mouth opening are several perioral buccal dilatators. These are striated and extend from the pharynx into the ring canal where they attach to the outer lophophoral base below the median axis of each tentacle (Fig. 10a, b, d, g). Two sets of abfrontal lophophoral base muscles are located distal to the insertion site at the abfrontal side of the lophophoral base,. The first is a short longitudinal muscle band with the second forming two lateral elements on its distal margin (i.e. tips of the branches of a ‘v’, in accordance to their designation as ‘v’-shaped muscles, cf. [9, 10]). On the frontal side of the lophophoral base are separate, smooth and circular muscle fibres at the base between each pair of tentacles, the basal transversal muscles (Fig. 10).

Each tentacle possesses two longitudinal striated muscles on the abfrontal and on the frontal side of each tentacle. The abfrontal bundle starts directly at the lophophoral base, whereas the frontal tentacle muscles appear more distally and show a significant gap towards the lophophoral base (Fig. 10e). Delicate circular muscle fibers were observed on the tentacle tips (Fig. 10f).

#### Digestive tract musculature

The pharynx epithelium consists of a prominent myoepithelium with striated contractile fibres embedded in the lateral membranes. Externally, densely arranged cross-striated circular muscles engulf the pharynx (Figs. 9a and 10a, d). These muscles continue into the esophagus and become slightly less dense and prominent. A few sparse longitudinal muscles are present in the lining of the esophagus (Fig. 9a). This arrangement of circular and few longitudinal fibres extends to the cardiac valve. The cardia and the caecum show only a sparse net of few smooth circular and longitudinal fibres (Fig. 9a). The pylorus lacks any distinct musculature, and the intestinal wall is lined solely by several smooth longitudinal muscle fibres (Fig. 9b).

### Autozooidal nervous system

#### Cerebral ganglion

**Fig. 11:**
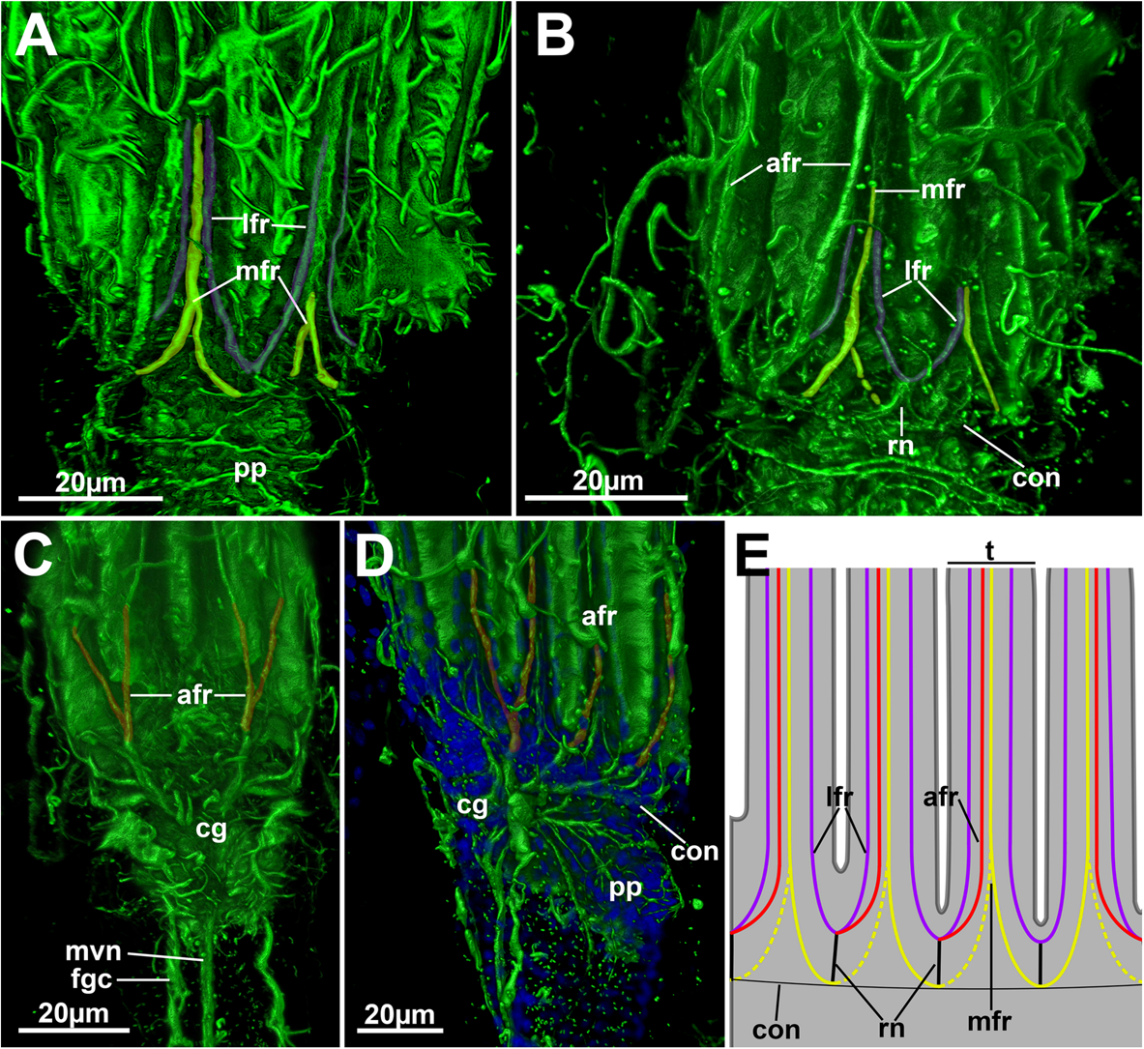
Tentacle innervation of *Hypophorella expansa*. Acetylated alpha tubulin staining (green), nuclei (blue), CSLM, volume rendering. **a**, **b** Two specimens showing the lophophoral base and highlighted medio-frontal neurite bundles (yellow) and latero-frontal ones (purple). **c** Anal view of the lophophoral base showing the two abfrontal neurite bundles (red) that split to innervate two adjacent pairs of tentacles above the ganglion. **d** Oblique view showing the abfrontal neurite bundles (red) on the anal and lateral sides of the lophophore. **e** Schematic drawing of the main tentacle neurite bundle branching. Latero-frontal neurites in purple, medio-frontal neurites in yellow and abfrontal neurites in red. Note that distinct asymmetries are present in the medio-frontal tentacle neurite bundle (indicated by dashed lines). Abbreviations: afr – abfrontal neurite bundle, cg – cerebral ganglion, con – circum-oral nerve ring, fgc – foregut ciliation, lfr – latero-frontal neurite bundle, mfr. – medio-frontal neurite bundle, mvn – medio-visceral neurite bundle, pp. – peripharyngeal plexus, rn – radial nerves

The center of the nervous system of an autozooid, the cerebral ganglion, is located at the anal side of the lophophoral base (Figs. 1, 5, 6a and 11c, d). It gives rise to a circum-oral nerve ring that runs around the lophophoral base to its oral side (Figs. 5c and 11d). Neurite bundles emerging directly from the ganglion innervate the tentacles (in combination with the circum-oral nerve ring), the tentacle sheath with two prominent tentacle sheath neurite bundles, and the foregut with several visceral neurite bundles (see below).

#### Tentacle innervation

In total four different neurite bundles innervate each tentacle. Three frontal neurite bundles (two latero-frontal and one medio-frontal) and a single abfrontal tentacle neurite bundle are present in *Hypophorella expansa* (Fig. 11). On the anal side, a pair of thicker neurite bundles emanate from the cerebral ganglion to the tentacles directly above the ganglion where they split into the respective tentacle bundles (Fig. 6a), whereas the tentacle neurite bundles emanate directly from the circum-oral nerve ring on the lateral and oral sides (Fig. 11). The roots of the latero-frontal and abfrontal neurite bundles emerge from radial nerves (intertentacular forks) that extend in an intertentacular position (Fig. 11e). From the latter, a single thicker abfrontal neurite bundle root emerges more proximally and always two, thinner latero-frontal bundles emanate more distally into adjacent tentacles. Only at the anal-most/adneural tentacles, the neurite bundles branch off two abfrontal neurite bundles to innervate the abfrontal side of two neighboring tentacles (Figs. 6a and 11c, d).

The medio-frontal neurite bundles originate from two proximal rootlets that emerge from an intertentacular position. In many observed specimens the roots were of asymmetrical thickness with one being thinner or in some cases even absent. The two rootlets run medially from adjacent radial nerves in distal direction where they join to form a single medio-frontal neurite bundle (Fig. 11a, b, e).

#### Tentacle sheath, apertural and parietal innervation

Two distinct thick neurite bundles (so-called ‘direct nerves’ of the tentacle sheath neurite bundles) emerge from the disto-lateral parts of the cerebral ganglion that project into the tentacle sheath (Figs. 5 and 6). More proximally the trifid nerve emerges from the ganglion, projects laterally and then splits into several branches. One of the branches turns distally into the tentacle sheath close to the lophophoral base (Fig. 5c and 6a) and terminates in this area.

The direct nerves project as tentacle sheath neurite bundle to the terminal end of the tentacle sheath into the direction of the diaphragm (Fig. 6b, c, e). On their distal traverse, they split at least once and one branch enters each duplicature band (Fig. 6c). Some neurite bundles project further to innervate the diaphragm and vestibular wall. The neurite bundles passing along the frontal duplicature band turn proximally once they reach the body wall and innervate the parietal muscles as parietal nerves (Fig. 6d, e).

#### Visceral innervation

Several neurite bundles emerge from the median and lateral proximal areas of the cerebral ganglion and converge on the anal side of the foregut to form the medio-visceral neurite bundle (Figs. 5 and 6a). Sometimes few bundles also remain as very thin bundles slightly laterally of the main neurite bundle. Additional latero-visceral bundles run in parallel to the medio-visceral bundle on the lateral anal side of the foregut. These emerge from the trifid nerve which projects from the proximo-lateral side on each side of the ganglion (Figs. 5c and 6a). From the same area as the trifid nerve, the roots of the peripharyngeal plexus emanate and surround the pharynx (Figs. 5d and 11d).

## Discussion

### Colony of *Hypophorella expansa*

The analysis of the housing tube of *Chaetopterus* sp. revealed a colonial structure of *Hypophorella expansa*, as previously described [7]. A stolon forms one single autozooid at most, which is laterally attached to the distal stolon capsule [7, 12]. The existence of kenozooidal stolons without attached autozooids is regarded as a form of division of labor [12]. The chief task of the main branches is growth and dispersal of the colony itself, whereas the task of the secondary branches is the formation of autozooids via budding [12]. *H. expansa* is placed inside the Walkerioidea, whose members possess autozooids either in median position (primary condition of creeping stolons) or in lateral position (secondary condition). Regarding the colony growth pattern, a laterally attached autozooid at the distal ends of the creeping stolons of *H. expansa* is therefore regarded as an autapomorphy. An additional autapomorphy of *H. expansa* concerning the growth pattern is that autozooids of succeeding stolons are attached in an alternating manner regarding their lateral position (if the autozooid of a preceding stolon is attached on the left side, the autozooid of the succeeding stolon is attached on the right side). An orthogonal branching of stolons probably results in an efficient dispersal of the colony to occupy a certain area and thus may facilitate a rapid colonization of the polychaete tube.

### Stolon structure

The peculiar wrinkling of the proximal part of an adult stolon of *Hypophorella expansa* is unique. A slightly comparable chitinous thickening of cystid walls is present in the autozooidal cystid walls in *Triticella flava* [13]. In the latter a chitinous hardening is present in the cystid wall of an autozooid [3]. In *H. expansa* there are numerous wrinkles that occur only in the slim proximal parts of the stolons following the wrinkle-free attachment sites where the pore plates are situated [7]. The presence of wrinkles, or chitinous hardenings in general, may provide higher stability to the cystid walls.

Autozooids and their corresponding stolons as well as the neighboring stolons are separated via a perforated septum that is formed during the budding process [7]. The observed cells surrounding these septa from both sides form a rosette, as previously reported [7], which supposedly acts in communication between stolons [14]. Previous assumptions concerning the presence of a closing membrane in these pores [7] can be rejected, since a similar cell complex around the septal perforation, in which “special cells” and “cincture cells” project through the perforation to the other side of the pore, as found in other gymnolaemates [14, 15], were observed. The rosette functions both as a mechanical plug and transport epithelium [14]. The rosettes of the vesicularioidean ctenostome *Bowerbankia*/*Amathia imbricata* were previously investigated in detail. As a member of the Vesicularioidea it possesses a well-developed funicular system. In *H. expansa* only a very delicate funiculus is present in its autozooids (Fig. 6d). In walkerioideans the funicular system is absent in the stolons ([3, 12], this study), contrary to previously reported funicular cords in stolons of *H. expansa* [7]. It appears highly likely that a detached body wall was previously misinterpreted as funicular cord within the cuticular lining of the stolon. Instead, a median transversal muscle is present in the distal stolon parts of the Walkerioidea (not present in Vesicularioidea), which functionally substitutes a stolonial funicular system (present in Vesicularioidea) presumably facilitating intracolonial transport. Such a muscle is also present during autozooidal development in the Paludicelloidea, which were reported previously considered closely related to the Walkerioidea [12].

The interrelationships of the ctenostome superfamilies remain obscure [1, 2]. In a recent phylogenetic analysis, the Walkerioidea are placed within a polytomy with Victorelloidea and Vesicularioidea as closest relatives [1]. This situation with lack of a funicular system and a present median transversal muscle in Walkerioidea versus a present funicular system and no median transversal muscle in Vesicularioidea shows alternative strategies with respect to effective transportation of nutrients inside stolons [16]. While the “cincture cells” in *Bowerbankia*/*Amathia* show thick and thin filaments the diameters of which resemble those of myofilaments [14], in the present study we did not detect f-actin in the observed equivalent cells of *H. expansa*. F-actin signal is present in the “special cells”, however, and they project medially through the pore.

### Gross autozooidal morphology

The observed autozooids confirm the general morphology reported by Ehlers [7]. They are vase-shaped, translucent and possess non-calcified, chitinous cystids. The two space balloons are positioned fronto-laterally on the distal part of the autozooids. Previously described perforations that connect the body cavity with the space balloons [8] were not observed in the present study. The cellular lining of the inner side of the space balloon cystid wall could be an ontogenetic rudiment that eventually dissolves once the nutrient reservoir is exhausted. Apart from the cellular lining, the spheres are filled with fluid. The structures themselves are regarded as buds that did not fully develop into autozooids [8] and the position also resembles the budding area (disto-lateral) of other gymnolaemates [17]. Regarding the environment of *Hypophorella expansa*, it can be assumed that multiple tube layers secreted by the polychaete worm increase the pressure on the autozooids and the entire colony. The presumed function of these space balloons for providing space and as mechanical protective structures thus appears sound [8]. In addition, the space balloons serve in providing space for unfolding the gnawing apparatus that otherwise would be dysfunctional. *Hypophorella expansa* is obviously adapted to the mechanical pressure created by the movement of the annelid inside its tube. The lophophore retraction process has to be maintained. Classical studies reported that an increasing mechanical pressure to the cystid walls lead to the inability of retraction [8].

The digestive tract of *H. expansa* is principally similar to those of other bryozoans (cf. [18]). Ciliation of the gut is restricted to the upper part of the pharynx and particularly the pylorus in gymnolaemate bryozoans. In addition, few, probably sensory, cilia are located in the epithelium of the foregut and project into the lumen of the gut. The cardia of *H. expansa* shows sparse ciliation in its linings and distinct ciliation at the cardiac valve. A delicate but consistent ciliation of the caecum, and a very dense and prominent ciliation of the pylorus and the proximal intestine are present. Not many bryozoans have been investigated with regard to the variation of cilia in the different parts; the present study suggests that there is probably more variation than previously expected.

The anus of *Hypophorella expansa* has a conspicuous position in the tentacle sheath, almost at the vestibular wall. Such an anal position is also found in several alcyonidioids [19, 20] and *Harmeriella terebrans* [21]. On the contrary, the anus is positioned more or less on the proximal half of the tentacle sheath, more closely to the mouth opening, in the vesicularioid *Amathia verticillata* [22], the alcyonidioid *Flustrellidra hispida* (as *Flustra hispida*, [23]), the hislopioid *Hislopia malayensis* [9] and the paludicellioid *Paludicella articulata* [24, 25]. From a parsimonious perspective, it seems that a proximally positioned anus reflects the ground pattern in ctenostomes, whereas the distal condition probably evolved at least twice independently (at least once in Alcyonidioidea and once in Walkerioidea). Functionally, a clearer distinction of the anus from the mouth is regarded as adaptation of fecal pellet disposal not interfering with suspension feeding [26].

### Apertural musculature and movement of the gnawing apparatus in *Hypophorella expansa*

In all bryozoans the distal end of the tentacle sheath is separated from the vestibular wall via a diaphragm, which possesses a diaphragmatic sphincter [10, 18]. The vestibular wall, diaphragm and distal tentacle sheath are associated with apertural muscles in all ctenostomes including *Hypophorella expansa* ([11], this study). In all bryozoans it consists of duplicature bands (parieto-vaginal bands) and prominent vestibular muscles [10]. Duplicature bands are reduced in many ctenostomes including most walkerioideans [10, 11]. *Hypophorella expansa* is one of the few walkerioideans that possesses distinct duplicature bands ([7], this study). Duplicature bands for *Farrella repens* were indicated, but not specifically mentioned (cf. [10]). Since these bands are present in the general bryozoan and also ctenostome bauplan [3, 11], it appears that these bands have been reduced several times in some clades. Indications from at least two species suggest that their absence is not a defining character of walkerioideans.

The remaining apertural muscles in most ctenostomes consists of four parieto-vestibular and parieto-diaphragmatic muscles, the former running from the body wall to the vestibular wall and the latter from the body wall to the area of the diaphragmatic sphincter. These muscles can be arranged radially or bilaterally in ctenostomes [10]. In contrast to other walkerioideans, the muscles in *Hypophorella expansa* show a strong asymmetry with the parieto-vestibular muscles shifted in distal direction and the parieto-diaphragmatic muscles located only laterally. Along with the prominence of these muscles, this corresponds to the operation and function of the gnawing apparatus and also correlates to colony morphology. Other walkerioideans show upright, erect autozooidal tubes with a radial arrangement of apertural muscles, whereas a bilateral arrangement is present in flat encrusting forms [10, 11] such as *Hyphophorella expansa*.

Not all of these muscles were previously recognized, and the traverse of some was wrongly interpreted [9]. The prominent lip-like frontal and basal circular fibres of the vestibular muscles are unique among bryozoans. In most bryozoans, vestibular wall muscles are sparse or even absent. Only alcyonioidid ctenostomes show a dense orifical sphincter [11]. The two bundles on the basal side reaching towards the gnawing apparatus (bov in Fig. 8) were previously unrecognized. Along with the parieto-vestibular muscles, these muscles are responsible for proper folding of the gnawing apparatus in retracted condition. Distinct musculature only associated with the gnawing apparatus allowing an autonomous movement is not present as previously indicated [9]. Instead, the entire apparatus is mainly moved by increased hydrostatic pressure effectuated by the parietal muscles. The basal parieto-vestibular muscles aid in the unfolding of the entire gnawing apparatus. The frontal ones are antagonists, and move it backwards during the retraction process. Prominent parieto-diaphragmatic muscles are necessary to rapidly withdraw the protruded diaphragmatic area prior to the complete infolding of the vestibular wall including the gnawing apparatus, otherwise the rapid retraction of the tissues would contact the teeth of the gnawing apparatus when the teeth-bearing vestibular wall are folded backwards.

### Remaining autozooidal musculature

Muscle systems of bryozoans and especially ctenostomes were recently summarized [11, 18]. With the exception of the apertural muscles, only few details will be discussed that add to the discussion already provided in Schwaha & Wanninger [11].

1. The retractor muscles are the most prominent muscles in bryozoans and occur as two bundles that originate from the proximal or lateral cystid wall and insert only at the lophophoral base in Gymnolaemata [18]. In *Hypophorella expansa*, few sparse bundles were also detected that insert at the foregut, a condition similar to other bryozoans (e.g. [27]). Only most recently a retractor muscle inserting at various points on the oral side of the gut was described in a ctenostome bryozoan [28]. It seems that there is some variability in the insertion areas of the retractor muscles (at least in ctenostomes).
  Concerning the nature of retractor muscle fibres as striated or smooth, both scenarios have been described (cf. [11]). The situation found in *Hypophorella expansa* is the first to show a mosaic of striated and smooth fibres in a bundle.
2. Tentacle sheath muscles of all Myolaemata (cf. [16, 18]) are generally longitudinal muscle fibres. A net of diagonally crossing fibres as observed in *Hypophorella expansa* is only present in victorellid and walkerioidean ctenostomes [11], which confirms, along with other characters, the affinity of this taxon to the Walkerioidea.
3. The lophophoral muscles are similar to other ctenostomes (cf. [11]). A distinct feature observed in *Hypophorella expansa* is the more distal appearance of the frontal tentacle muscles compared to the abfrontal ones that start at the lophophoral base. Such a gap has not been observed for any other ctenostome. Only in the cyclostome *Cinctipora elegans* a distinct gap is present, but for both, frontal and abfrontal tentacle muscles [29].

*Hypophorella expansa* possesses actin concentrations at their distal tentacle areas. Such a condition was only reported recently for the ctenostome *Hislopia malayensis* [10]. Sperm release has been previously reported through the tips of either the two anal-most tentacles of malacostegine cheilostomes or the tips of all tentacles in all other investigated cheilostomes [30, 31]. It is assumed that the actin signal represents a delicate muscular sphincter that facilitates closure of the tentacle lumen. Since *H. expansa* possesses distal muscular tips in each tentacle, presumably sperm release is conducted through the tentacles as well.

In *Hyphophorella expansa* the frontal circular lophophoral base muscles are present as circular basal transverse muscles. Other ctenostomes generally have two different variants of this muscle, as complete ring of circular muscles or as separate, intertentacular fibre bundles, as in *H. expansa*. Some species even show an intermediate of an almost complete ring with only thin fibres at tentacular positions [11]. In all other analysed walkerioideans, this muscle is present as continuous or almost complete ring at the lophophoral base. *Hypophorella expansa* is thus the first walkerioidean to show distinct basal transversal muscles.

### Nervous system

The bryozoan nervous system was analysed and discussed in several recent studies (e.g. [18, 24, 29, 32–34]). Accordingly, only the major differences are discussed in detail here.

The main neural features including the cerebral ganglion are identical to most other bryozoans [35]. Main differences are present in the tentacle and tentacle sheath innervation.

1) The reported tentacle innervation pattern for Gymnolaemata with one abfrontal, one medio-frontal and one pair of latero-frontal neurite bundles [35] is also found in *Hypophorella expansa*. All mentioned tentacle neurite bundles are located basiepithelially. The branching of the latero-frontal neurite bundles from the intertentacular radial nerves is present in all bryozoans and does not show any difference to other bryozoans (see [18, 24, 32, 33, 35]). Likewise, the abfrontal tentacle neurite bundle shows a similar pattern in phylactolaemate [36, 37], cyclostome [29, 33, 34] and ctenostome bryozoans [24, 33, 38]. In most descriptions two roots arise from both radial nerves and merge medially to form a single neurite bundle. In cheilostome bryozoans, the abfrontal bundle is usually considered to emanate directly from the circum-oral nerve ring or ganglion and not from an intertentacular area [38, 39]. However, there are indications that these also originate intertentacularly, but are always asymmetrical emerging only from one side, which has probably been interpreted as direct origin (cf. [18]). Such an asymmetric pattern is also present in *Hypophorella expansa* and other bryozoans [29, 36].

The medio-frontal neurite bundle originates directly from the circum-oral nerve ring in other gymnolaemates (cf. [24]) and one cyclostome [29], and intertentacularly from the radial nerves in phylactolaemates [36, 37]. Two lateral roots emerging from the circum-oral nerve ring that medially merge were described for the cyclostome *Crisia* [33, 34] and is also present in *Hypophorella expansa*. This indicates that an intertentacular origin might also have been present in the ground pattern of myolaemate bryozoans.

2) The tentacle sheath of gymnolaemate bryozoans is innervated by two compound tentacle sheath neurite bundle that are comprised of two bundles merging shortly after their emergence from the cerebral ganglion (cf. [35, 39]). One of them projects directly from the disto-lateral sides of the cerebral ganglion, as direct nerve, into the tentacle sheath. The second one emerges from a branch of the trifid nerve, which projects more proximally from the cerebral ganglion. In *Hypophorella expansa*, only the direct nerves innervate the tentacle sheath, whereas the trifid nerve branches off a neurite bundle in direction of the tentacle sheath similar to other gymnolaemates, but it terminates soon after and does not merge with the direct nerve. A tentacle sheath innervation with a single pair, from a similar location as the direct nerves, was found in the cyclostome *C. elegans* [28].

### Reproductive pattern of *Hypophorella expansa*

Bryozoan reproductive patterns can be classified into different categories mainly depending on oogenesis, amount of developing oocytes and presence of absence of embryonic incubation (cf. [40, 41]). Accordingly, *Hypophorella expansa* belongs to first reproductive pattern, i.e. broadcasting [41, 42]. Broadcasting is less common among Bryozoa, whereas most species (including ctenostomes) incubate their embryos (cf. [41, 43–45]). Broadcasting in *H. expansa* includes the production of many small oligolecithal oocytes and a presumed feeding larva, the cyphonautes [7, 9]. Most broadcasting bryozoans release fertilized egg via an intertentacular organ, whereas three broadcasting ctenostomes, *H. expansa*, *Farrella repens* and *Hislopia malayensis* release eggs via a supraneural coelomopore [41], which presumably is an ancestral pattern.

Male and female gonads were previously stated to occur in the same zooid [7]. Only gonochoristic zooids were found in this similar to other bryozoans where non-simultaneous gonad maturation is common [41]. In addition, paired testes were reported [7], which we cannot support in the current study.

## Conclusions

The present study analyses the peculiar and little known boring ctenostome *Hypophorella expansa*. Its particular lifestyle and habitat in polychaete tube walls necessitated several morphological adaptations. Space balloons on the fronto-lateral side of the autozooids provide space between successively secreted tube layers of the polychaete host. The stolons possess a median transversal muscle in their distal capsules and a peculiar wrinkling at the inner surface of their ectocysts proximally to them. The wrinkles project inside the stolon lumen and may provide mechanical stability owing to the thinness of the stolons. The growth pattern of *H. expansa* may be an efficient way to colonize the space in between the layers of the polychaete tube. Since only one lateral autozooid is attached to a distal stolon capsule, the successive distal and lateral stolons allow a fast two dimensional dispersal of the colony. Autozooids of *H. expansa* possess a mechanical gnawing apparatus, which is integrated into their vestibular wall and associated with the apertural musculature. The gnawing procedure is linked to the protrusion–retraction mechanism of the polypide and is strongly associated with the very prominent apertural muscles, since no musculature is present to perform autonomous movements of the gnawing apparatus.

Interestingly, a similar association of gnawing structures and the vestibular wall was reported for the ctenostome *Harmeriella terebrans*, which bores in calcified cheilostome cystid walls. These two species are probably closely related and share a similar colony morphology [38]. Unfortunately, the latter has never been re-encountered after its discovery, as was the case for *H. expansa* for a long period. A reinvestigation and comparison of boring bryozoans in general using state of the art methods is required to assess morphological adaptations and how they may be linked to their mechanically boring life-style.

## Additional file

Additional file 1: **Video S1.** Detail of an autozooid of *Hypophorella expansa* showing movements of the polypide, including eversion and retraction of the vestibular wall including the gnawing apparatus. (MOV 455936 kb)

## Data Availability

The datasets and/or analyses from the current study are available from the corresponding author on reasonable request.

## References

[1] Todd JA, Herrera Cubilla A, Jackson JBC (2000). The central role of ctenostomes in bryozoan phylogeny. Proceedings of the 11th international Bryozoology association conference.

[2] Waeschenbach A, Taylor PD, Littlewood DTJ (2012). A molecular phylogeny of bryozoans. Mol Phylogenet Evol.

[3] Schwaha T. Ctenostomata. In: Schwaha T, editor. Handbook of Zoology Bryozoa. Berlin: de Gruyter. in press.

[4] Bock P, Gordon DP (2013). Phylum Bryozoa Ehrenberg, 1831. Zootaxa.

[5] Pohowsky RA, Pouyet S (1975). Boring Bryozoa. Bryozoa 1974.

[6] Pohowsky RA (1978). The boring Ctenostomate Bryozoa: taxonomy and paleobiology based on cavities in calcareous substrata. Bull Ame Paleontology.

[7] Ehlers E (1876). *Hypophorella expansa*, ein Beitrag zur Kenntnis der minierenden Bryozoen. Abhandlungen der Koeniglichen Gesellschaft der Wissenschaften zu Goettingen.

[8] Joyeux-Laffuie J (1888). Description du *Delagia chaetoptei* (J. J.-L.). Arch Zool Exp Gen 2 Serie.

[9] Prouho H (1892). Contribution à l’histoire des Bryozaires. Arch Zool Exp Gen (2nd series).

[10] Schwaha T, Wood TS, Wanninger A (2011). Myoanatomy and serotonergic nervous system of the ctenostome *Hislopia malayensis*: evolutionary trends in bodyplan patterning of Ectoprocta. Front Zool.

[11] Schwaha T, Wanninger A (2018). Unity in diversity: a survey of muscular systems of ctenostome Gymnolaemata (Lophotrochozoa, Bryozoa). Front Zool.

[12] Jebram D (1973). Stolonen-Entwicklung und Systematik bei den Bryozoa Ctenostomata. Z Zool Syst Evol.

[13] Hayward PJ: Ctenostome Bryozoans. London, Leiden, Köln, Kobenhavn: E.J. Brill/Dr.W. Backhuys for The Linnean Society of London & The Estuarine and Brackish-Water Siences Association.; 1985.

[14] Gordon DP, Pouyet S (1975). Ultrastructure of communication pore areas in two bryozoans. Bryozoa, 1974.

[15] Mukai H, Terakado K, Reed CG, Harrison FW, Woollacott RM (1997). Bryozoa. Microscopic anatomy of invertebrates.

[16] Schwaha T, Ostrovsky AN, Wanninger A: Key novelties in the evolution of aquatic colonial phylum Bryozoa: Evidence from soft body morphology. submitted. 10.1111/brv.12583 PMC7317743 32032476

[17] Jebram D (1986). The ontogenetical and supposed phylogenetical fate of the parietal muscles in the Ctenostomata (Bryozoa). Z zool Syst Evol.

[18] Schwaha T. Morphology of bryozoans. In: Schwaha T, editor. Handbook of Zoology: Bryozoa. Berlin: DeGruyter. in press.

[19] Silbermann S (1906). Untersuchungen über den feineren Bau von *Alcyonidium mytili*. Arch f Naturg.

[20] Ryland JS, Porter JS (2006). The identification, distribution and biology of encrusting species of *Alcyonidium* (Bryozoa: Ctenostomatida) around the coasts of Ireland. Biol Environ.

[21] Borg F (1940). On the genus *Tubiporella* and a new boring bryozoan. Zool Bidr Uppsala.

[22] Gerwerzhagen A. Untersuchungen an Bryozoen. Sitzungs Heidelb Akad Wiss Math Nat Kl Abt B. 1913;9:1–16.

[23] Graupner H (1930). Zur Kenntnis der feineren Anatomie der Bryozoen. Z Wiss Zool.

[24] Weber A, Wanninger A, Schwaha T (2014). The nervous system of *Paludicella articulata* - first evidence of a neuroepithelium in a ctenostome ectoproct. Front Zool.

[25] Schwaha TF, Wanninger A (2015). The serotonin-lir nervous system of the Bryozoa (Lophotrochozoa): a general pattern in the Gymnolaemata and implications for lophophore evolution of the phylum. BMC Evol Biol.

[26] McKinney MJ (1997). Fecal pellet disposal in marine bryozoans. Invertebr Biol.

[27] Gawin N, Wanninger A, Schwaha T (2017). Reconstructing the muscular ground pattern of phylactolaemate bryozoans: first data from gelatinous representatives. BMC Evol Biol.

[28] Schwaha T, Edgcomb VP, Bernhard JM, Todaro MA. *Aethozooides uraniae*, a new deep sea genus and species of solitary bryozoan from the Mediterranean with a revision of the Aethozoidae: Marine Biodiversity. in press

[29] Schwaha TF, Handschuh S, Ostrovsky AN, Wanninger A. Morphology of the bryozoan *Cinctipora elegans* (Cyclostomata, Cinctiporidae) with first data on its sexual reproduction and the cyclostome neuro-muscular system. BMC Evol Biol. 2018;18. 10.1186/s12862-018-1206-1 PMC6000935 29898669

[30] Silen L (1966). On the fertilization problem in the Gymnolaematous Bryozoa. Ophelia.

[31] Silen L (1972). Fertilization in the Bryozoa. Ophelia.

[32] Temereva EN, Kosevich IA (2016). The nervous system of the lophophore in the ctenostome *Amathia gracilis* provides insight into the morphology of ancestral ectoprocts and the monophyly of the lophophorates. BMC Evol Biol.

[33] Temereva EN, Kosevich IA (2018). The nervous system in the cyclostome bryozoan *Crisia eburnea* as revealed by transmission electron and confocal laser scanning microscopy. Front Zool.

[34] Worsaae K, Frykman T, Nielsen C. The neuromuscular system of the cyclostome bryozoan *Crisia eburnea* (Linnaeus, 1758). Acta Zool. 2019.

[35] Gruhl A, Schwaha T, Schmidt-Rhaesa A, Harzsch S, Purschke G (2015). Bryozoa. Structure and evolution of invertebrate nervous system.

[36] Ambros M, Wanninger A, Schwaha T (2018). Neuroanatomy of the plumatellid bryozoan *Hyalinella punctata* reveals a common pattern in a small group of freshwater bryozoans. J Morphol.

[37] Shunkina KV, Zaytseva OV, Starunov VV, Ostrovsky AN (2015). Comparative morphology of the nervous system in three phylactolaemate bryozoans. Front Zool.

[38] Schwaha T, Wood TS (2011). Organogenesis during budding and lophophoral morphology of *Hislopia malayensis* Annandale, 1916 (Bryozoa, Ctenostomata). BMC Dev Biol.

[39] Lutaud G, Woollacott RM, Zimmer RL (1977). The bryozoan nervous system. Biology of bryozoans.

[40] D'Hondt JL (1983). Tabular keys for identification of the recent Ctenostomatous Bryozoa. Mémoires de L'Institut Océanographique. Monaco.

[41] Ostrovsky AN (2013). Evolution of sexual reproduction in marine invertebrates: example of gymnolaemate bryozoans.

[42] Reed CG, Giese AC, Pearse JS, Pearse VB (1991). Bryozoa. Reproduction of marine invertebrates VI echinoderms and Lophophorates.

[43] Ostrovsky AN, Gordon DP, Lidgard S (2009). Independent evolution of matrotrophy in the major classes of Bryozoa: transitions among reproductive patterns and their ecological background. Mar Ecol Prog Ser.

[44] Nekliudova UA, Schwaha TF, Kotenko ON, Gruber D, Cyran N, Ostrovsky AN (2019). Sexual reproduction of the placental brooder *Celleporella hyalina* (Bryozoa, Cheilostomata) in the White Sea. J Morphol.

[45] Schwaha T, Moosbrugger M, Walzl M, Ostrovsky AN (2019). First ultrastructural evidence of placental nutrition in a ctenostome bryozoan: example of Amathia verticillata. Zoomorphology.

